# The impact of cigarette smoking on life expectancy in schizophrenia, schizoaffective disorder and bipolar affective disorder: An electronic case register cohort study

**DOI:** 10.1016/j.schres.2021.09.006

**Published:** 2021-12

**Authors:** Edward Chesney, Deborah Robson, Rashmi Patel, Hitesh Shetty, Sol Richardson, Chin-Kuo Chang, Philip McGuire, Ann McNeill

**Affiliations:** a King's College London, Department of Psychosis Studies, Institute of Psychiatry, Psychology & Neuroscience, London, UK; b King's College London, Addictions Department, Institute of Psychiatry, Psychology & Neuroscience, London, UK; c SPECTRUM Consortium, UK; d South London and Maudsley NHS Foundation Trust, Biomedical Research Centre Nucleus, London, UK; e Global Health Program, College of Public Health, National Taiwan University, Taipei City, Taiwan; f King's College London, Department of Psychological Medicine, Institute of Psychiatry, Psychology & Neuroscience, UK

**Keywords:** Tobacco, Smoking, Mortality, Life expectancy, Bipolar affective disorder, Schizophrenia

## Abstract

Severe mental disorders are associated with a life expectancy that is 10–20 years shorter than the general population's. The prevalence of cigarette smoking in these populations is very high. We examined the effect of smoking on life expectancy and survival in patients with a diagnosis of schizophrenia, schizoaffective disorder or bipolar affective disorder from 2007 to 2018 in South East London, UK. Smoking status was determined using unstructured text data extracted from electronic health records. A total of 21,588 patients were identified of which 16,717, (77.4%) were classified as current smokers and 3438 (15.9%) as non-smokers. In female participants, life expectancy at birth was 67.6 years in current smokers (95% CI: 66.4–68.8) and 74.9 years in non-smokers (95% CI: 72.8–77.0), a difference of 7.3 years. In male participants, life expectancy at birth was 63.5 years in current smokers (95% CI: 62.5–64.5) and 68.5 years in non-smokers (95% CI, 64.4–72.6), a difference of 5.0 years. Adjusted survival models found that current smoking status was associated with an increased mortality risk for both females (aHR: 1.42, 95% CI: 1.21–1.66, *p* < 0.001) and males (aHR: 1.49; 95% CI: 1.25–1.79, *p* < 0.001). In terms of the effect sizes, these risks were similar to those associated with a diagnosis of co-morbid alcohol or opioid use disorder. Smoking may account for a substantial proportion of the reduced life expectancy in patients with psychotic disorders. Increased emphasis on reducing cigarette smoking in these populations may be the most effective way to reduce the mortality gap with the general population.

## 1 Introduction

Mental disorders are associated with an increased risk of mortality and reduced life expectancy (Chesney et al., 2014). For schizophrenia, schizoaffective disorder and bipolar affective disorder, life expectancy at birth is around 10–20 years shorter than the general population's (Chang et al., 2011; Chesney et al., 2014). The size of this difference is alarming and recent evidence from the UK suggests that mortality risks may be increasing (Hayes et al., 2017). Various explanations have been proposed and include increased suicide and accidental death (Chesney et al., 2014), poor provision of and engagement with physical and mental healthcare (De Hert et al., 2011), and increased rates of alcohol, illicit substance use and cigarette smoking (Toftdahl et al., 2016).

Smoking may be of particular importance as its prevalence is so high. In schizophrenia, meta-analyses estimate that over half of people with the disorder smoke (De Leon and Diaz, 2005; Mitchell et al., 2013). In the UK, around 45% of people with a diagnosis of schizophrenia and 37% with bipolar affective disorder are smokers, levels which are twice as high as found in the rest of the population (Royal College of Physicians; Royal College of Psychiatrists, 2013). As a result of these differences, they are much more likely to die from smoking-related diseases. The standardised mortality ratios for tobacco-related diseases are 2.45 (95% CI: 2.41–2.48) in schizophrenia and 1.57 (95% CI: 1.53–1.62) in bipolar affective disorder (Callaghan et al., 2014).

In the general population, smokers have a life expectancy which is 7–10 years shorter than non-smokers (Al Mamun et al., 2004; Doll et al., 1994; Sakata et al., 2012). To our knowledge, the effect of smoking on life expectancy in those with a diagnosed mental disorder has never been investigated before. One study from the United States examined life expectancy in those with ‘serious psychological distress’ (SPD), measured using the self-reported Kessler scale, with a score of ≥13/24 used as a marker for serious mental illness (Tam et al., 2016). It estimated that people with SPD who also smoke have a life expectancy 14.9 years shorter than never smokers without SPD. In comparison, never smokers with SPD had a life expectancy only 5.3 years shorter. The authors suggested that, even after taking into account confounding variables, a large part of the 15-year life expectancy gap, up to two-thirds, could be attributed to smoking.

Quantifying the contribution of cigarette smoking to reduced life expectancy in people diagnosed with these disorders will help clarify the potential impact of smoking cessation interventions (Ilyas et al., 2017). It also aids those aiming to allocate resources cost-effectively and equitably. In this study, we therefore aimed to estimate life expectancy at birth according to smoking status in people with a clinical diagnosis of either schizophrenia, schizoaffective disorder or bipolar affective disorder using data from a large electronic secondary mental healthcare database. To assess the impact of potential confounding factors, we planned survival analyses incorporating a broad range of demographic, socioeconomic and clinical variables.

## 2 Material and methods

### 2.1 Data extraction

We obtained data from the South London and Maudsley NHS Foundation Trust (SLaM) Biomedical Research Centre (BRC) Case Register. The register contains fully anonymized electronic health records of over 400,000 patients receiving secondary mental healthcare in South East London. The records include both structured and unstructured (free text) fields from case notes, correspondence and other assessments. Data were extracted from the case register using the Clinical Record Interactive Search (CRIS) system. Information on the case register and the CRIS system have been described elsewhere (Perera et al., 2016; Stewart et al., 2009).

The observation period was defined as 1st January 2007 to 31st December 2018. The study population included all patients who received an SMI diagnosis during the observation period, reached the age of 15 years before its end and had information on smoking status. SMI diagnoses included schizophrenia, schizoaffective disorder and bipolar affective disorder, as per the NHS England definition (NHS England, 2018). Diagnoses (assigned by clinicians according to International Classification of Diseases 10th Revision [ICD-10] criteria) were extracted from both structured and unstructured fields at the earliest recorded date during the observation period. Structured fields were used to obtain data on date of birth, sex, ethnicity, marital status, co-morbid diagnoses of any personality disorder (ICD-10: F61, F62), alcohol use disorder (ICD-10: F10), opioid use disorder (ICD-10: F11), or other substance use disorder (ICD-10: F12-F19, excluding tobacco use disorders), and admissions to a psychiatric hospital during the observation period. Index of Multiple Deprivation (IMD) scores were also obtained from structured fields. IMD is a widely used UK measure of deprivation which includes seven domains: income, employment, education, health, crime, housing and services, and living environment (Department for Communities and Local Government, 2011). IMD scores were grouped into quintiles. Deaths were identified using the NHS Care Records Service, a nationwide service that records every death in the UK once a formal death certificate has been issued.

### 2.2 Classification of smoking status

The CRIS-IE-Smoking application was used to obtain data on smoking status. The application uses natural language processing software to classify information on smoking from free-text fields into three categories: ‘current smoker’, ‘past smoker’, and ‘non-smoker’, with smoking of substances other than tobacco (e.g. cannabis and cocaine) excluded (Wu et al., 2013). When applied to 300 random documents with any key word about smoking, the application has demonstrated a precision (positive predictive value) of 80% and an annotation-level recall (sensitivity) of 88%.

Many individuals had multiple instances of smoking status data extracted, some of which conflicted. Each individual was therefore classified according to their most frequently recorded smoking status. Individuals without a single most frequent smoking status and those whose most frequently recorded status was ‘past smoker’ were excluded from further analyses. To assess the accuracy of this strategy, 200 randomly selected participants (100 current smokers and 100 non-smokers) had their case-notes reviewed by a psychiatrist [EC] blinded to their assigned smoking status. We then calculated the positive predictive values of ‘current smoker’ and ‘non-smoker’ categories.

### 2.3 Life expectancy estimation

We calculated life expectancy at birth for each gender according to smoking status using Chiang's method of abridged life tables (Chiang, 1984; Toson and Baker, 2003). For each individual, an ‘at-risk’ period was defined as the time between first ever diagnosis of the psychotic disorder or the start of the observation period (whichever occurs last), and end of the observation period or death (whichever occurs first). The total person-years at risk and the number of deaths within each five-year age band were then calculated. Data were not extracted for age bands below 15 years and it was assumed that at this age mortality risks are equal to general population risk. Instead, for the age bands 0–1 year, 1–4 years, 5–9 years and 10–14 years, mortality for the general population of England and Wales in 2012 (the mid-point of the observation period) were imputed (Office of National Statistics, 2012). In age bands with no deaths, general population death rates were imputed to prevent underestimation of the standard error. Life expectancy estimates were also calculated according to SMI diagnosis.

### 2.4 Survival analysis

Cox regression models for each gender were used to estimate crude and adjusted mortality hazard ratios (aHRs) for potential confounding factors. ‘Time to death’ was defined as the period from first ever diagnosis of psychotic disorder or the start of the observation period (whichever occurred last) until the date of death or censored at 31st December 2018. Missing data for ethnicity, marital status and deprivation quintiles were addressed using multiple imputation by chained equations under the assumption that missing observations were missing at random (Royston, 2004). We generated 25 imputed datasets (after 10 burn-in iterations) based on Monte Carlo errors estimated for each model parameter. The first model included age band as the only covariate. A second model included all variables: age band, ethnicity, marital status, deprivation, primary psychotic disorder diagnosis, admission to hospital and co-morbid personality, alcohol use, opioid use and other substance use disorder diagnoses. The proportional hazards assumption for the primary exposure variable, smoking status, was tested using a time interaction term. While we found no evidence that the effect of smoking status on mortality changed linearly with time for female participants (suggesting the proportional hazards assumption was met), this was not the case for males.

Stata (version 15.1) was used for all analyses apart from the life expectancy estimates which were completed using Microsoft Excel life tables downloaded from the ONS (Office for National Statistics, 2017a).

### 2.5 Ethical approval

The SLaM BRC Case Register and CRIS have received ethical approval as an anonymised data set for secondary analyses from the Oxfordshire Research Ethics Committee C (08/H0606/71 + 5).

## 3 Results

We identified 21,588 patients with a diagnosis of schizophrenia, schizoaffective disorder or bipolar affective disorder, who had reached the age of 15 and had smoking status data. Past-smokers (470 [2.2%]) and individuals with an unclear smoking status (963 [4.5%]) were excluded. The final study population included 20,155 participants, of whom 16,717 (82.9%) were current smokers and 3438 (17.1%) were non-smokers. The positive predictive value of the most frequently recorded smoking status was 88% for ‘current smoker’ and 87% for ‘non-smoker’. The demographic and clinical characteristics of the population are described in Table 1, Table 2.

**Table 1:** Female study population: demographic and clinical characteristics and results of the survival analyses (*n* = 8993).

	All	Participants (n, %)	Current smokers (n, %)	Deaths (n, %)	Age-adjusted HR (95% CI)	*p* value	Adjusted HR (95% CI)	*p* value
		8993 (100%)	6927 (77.0%)	977 (10.9%)				
Age	<15	135 (1.5%)	119 (88.1%)	1 (0.7%)	0.19 (0.03–1.34)	0.095	0.19 (0.03–1.36)	0.098
	15–25	1487 (16.5%)	1253 (84.3%)	23 (1.5%)	0.36 (0.23–0.57)	<0.001	0.35 (0.23–0.56)	<0.001
	25–35	1890 (21.0%)	1479 (78.3%)	52 (2.8%)	0.52 (0.38–0.72)	<0.001	0.51 (0.37–0.71)	<0.001
	35–45	2028 (22.6%)	1568 (77.3%)	128 (6.3%)	Reference	–	Reference	–
	45–55	1587 (17.6%)	1235 (77.8%)	157 (9.9%)	1.59 (1.26–2.01)	<0.001	1.57 (1.24–1.98)	<0.001
	55–65	875 (9.7%)	640 (73.1%)	190 (21.7%)	3.55 (2.83–4.44)	<0.001	3.53 (2.79–4.46)	<0.001
	65+	991 (11.0%)	633 (63.9%)	426 (43.0%)	9.89 (8.11–12.06)	<0.001	10.32 (8.27–12.89)	<0.001
Ethnicity	White	4685 (52.1%)	3794 (81.0%)	619 (13.2%)	Reference	–	Reference	–
	Black Caribbean	959 (10.7%)	729 (76.0%)	147 (15.3%)	0.77 (0.64–0.92)	0.005	0.77 (0.64–0.93)	0.006
	Black African	1887 (21.0%)	1384 (73.3%)	123 (6.5%)	0.77 (0.63–0.94)	0.011	0.79 (0.64–0.97)	0.027
	South Asian	233 (2.6%)	143 (61.4%)	23 (9.9%)	0.67 (0.44–1.01)	0.055	0.74 (0.49–1.12)	0.156
	Other^a^	690 (7.7%)	506 (73.3%)	39 (5.7%)	0.75 (0.55–1.04)	0.086	0.79 (0.57–1.09)	0.156
	Not known	539 (6.0%)	371 (68.8%)	26 (4.8%)	–	–	–	–
Marital status	Single	5275 (58.7%)	4288 (81.3%)	426 (8.1%)	Reference	–	Reference	–
	Married	1380 (15.3%)	922 (66.8%)	159 (11.5%)	0.88 (0.73–1.05)	0.162	0.94 (0.78–1.14)	0.516
	Separated	1504 (16.7%)	1098 (73.0%)	352 (23.4%)	0.97 (0.83–1.14)	0.745	1.03 (0.88–1.20)	0.719
	Not known	834 (9.3%)	619 (74.2%)	40 (4.8%)	–	–	–	–
Deprivation	1st (High)	1443 (16.0%)	1050 (72.8%)	144 (10.0%)	Reference	–	Reference	–
	2nd	5814 (64.7%)	4480 (77.1%)	647 (11.1%)	0.99 (0.83–1.19)	0.955	0.99 (0.83–1.19)	0.937
	3rd–5th (Low)	1359 (15.1%)	1070 (78.7%)	152 (11.2%)	1.05 (0.84–1.32)	0.648	1.05 (0.84–1.33)	0.661
	Not known	377 (4.2%)	327 (86.7%)	34 (9.0%)	–	–	–	–
Primary diagnosis	Schizophrenia	4895 (54.4%)	3753 (76.7%)	632 (12.9%)	Reference	–	Reference	–
	Schizoaffective disorder	521 (5.8%)	408 (78.3%)	61 (11.7%)	0.97 (0.74–1.26)	0.793	0.96 (0.74–1.25)	0.748
	Bipolar affective disorder	3577 (39.8%)	2766 (77.3%)	284 (7.9%)	0.93 (0.81–1.07)	0.296	0.88 (0.76–1.02)	0.092
Psychiatric admission	No	5159 (57.4%)	3798 (73.6%)	516 (10.0%)	Reference	–	Reference	–
	Yes	3834 (42.6%)	3129 (81.6%)	461 (12.0%)	0.99 (0.87–1.12)	0.849	0.99 (0.87–1.13)	0.904
Personality disorder	No	7777 (86.5%)	5843 (75.1%)	922 (11.9%)	Reference	–	Reference	–
	Yes	1216 (13.5%)	1084 (89.1%)	55 (4.5%)	0.97 (0.74–1.27)	0.805	0.84 (0.64–1.11)	0.226
Alcohol use disorder	No	8482 (94.3%)	6447 (76.0%)	921 (10.9%)	Reference	–	Reference	–
	Yes	511 (5.7%)	480 (93.9%)	56 (11.0%)	1.52 (1.16–2.00)	0.002	1.30 (0.98–1.72)	0.074
Opioid use disorder	No	8791 (97.8%)	6726 (76.5%)	954 (10.9%)	Reference	–	Reference	–
	Yes	202 (2.2%)	201 (99.5%)	23 (11.4%)	2.51 (1.65–3.81)	<0.001	1.76 (1.10–2.82)	0.018
Other substance use disorder	No	8392 (93.3%)	6339 (75.5%)	933 (11.1%)	Reference	–	Reference	–
	Yes	601 (6.7%)	588 (97.8%)	44 (7.3%)	1.81 (1.32–2.48)	<0.001	1.42 (0.99–2.03)	0.056
Current smoker	No	2066 (23.0%)	0 (0.0%)	206 (10.0%)	Reference	–	Reference	–
	Yes	6927 (77.0%)	6927 (100%)	771 (11.1%)	1.51 (1.30–1.77)	<0.001	1.42 (1.21–1.66)	<0.001

a Other ethnicity includes: Chinese, Any other ethnic group, Any other mixed background and White and Asian categories.

**Table 2:** Male study population: demographic and clinical characteristics and results of the survival analyses (*n* = 11,162).

	All	Participants (n, %)	Current smokers (n, %)	Deaths (n, %)	Age-adjusted HR (95% CI)	*p* value	Adjusted HR (95% CI)	*p* value
		11,162 (100%)	9790 (87.7%)	1307 (11.7%)				
Age	<15	118 (1.1%)	99 (83.9%)	0 (0.0%)	–	–	–	–
	15–25	1944 (17.4%)	1745 (89.8%)	57 (2.9%)	0.46 (0.34–0.61)	<0.001	0.49 (0.37–0.66)	<0.001
	25–35	2774 (24.9%)	2484 (89.5%)	142 (5.1%)	0.7 (0.57–0.87)	0.001	0.72 (0.58–0.89)	0.002
	35–45	2881 (25.8%)	2560 (88.9%)	237 (8.2%)	Reference		Reference	–
	45–55	1850 (16.6%)	1609 (87,0%)	269 (14.5%)	1.9 (1.59–2.26)	<0.001	1.83 (1.53–2.19)	<0.001
	55–65	916 (8.2%)	786 (85.8%)	255 (27.8%)	3.83 (3.21–4.57)	<0.001	3.51 (2.92–4.23)	<0.001
	65+	679 (6.1%)	507 (74.7%)	347 (51.1%)	9.82 (8.32–11.6)	<0.001	9.67 (8.08–11.6)	<0.001
Ethnicity	White	5512 (49.4%)	4868 (88.3%)	847 (15.4%)	Reference	–	Reference	–
	Black Caribbean	1193 (10.7%)	1058 (88.7%)	166 (13.9%)	0.73 (0.62–0.86)	<0.001	0.73 (0.61–0.86)	<0.001
	Black African	2686 (24.1%)	2379 (88.6%)	160 (6.0%)	0.62 (0.52–0.74)	<0.001	0.64 (0.53–0.76)	<0.001
	South Asian	301 (2.7%)	236 (78.4%)	35 (11.6%)	0.76 (0.54–1.07)	0.111	0.82 (0.58–1.15)	0.243
	Other^a^	758 (6.8%)	667 (88.0%)	55 (7.3%)	0.78 (0.59–1.03)	0.079	0.81 (0.61–1.06)	0.128
	Not known	712 (6.4%)	582 (81.7%)	44 (6.2%)	–	–	–	–
Marital status	Single	8274 (74.1%)	7395 (89.4%)	900 (10.9%)	Reference	–	Reference	–
	Married	974 (8.7%)	749 (76.9%)	139 (14.3%)	0.8 (0.67–0.97)	0.019	0.92 (0.76–1.11)	0.364
	Separated	926 (8.3%)	797 (86.1%)	209 (22.6%)	0.96 (0.82–1.13)	0.656	1.02 (0.87–1.19)	0.827
	Not known	988 (8.9%)	849 (85.9%)	59 (6.0%)	–	–	–	–
Deprivation	1st (High)	1555 (13.9%)	1290 (83.0%)	175 (11.3%)	Reference	–	Reference	–
	2nd	7047 (63.1%)	6217 (88.2%)	837 (11.9%)	0.95 (0.81–1.12)	0.561	0.95 (0.81–1.13)	0.582
	3rd–5th (Low)	1632 (14.6%)	1442 (88.4%)	224 (13.7%)	1.1 (0.90–1.35)	0.333	1.1 (0.90–1.35)	0.334
	Not known	928 (8.3%)	841 (90.6%)	71 (7.7%)	–	–	–	–
Primary diagnosis	Schizophrenia	8147 (73.0%)	7244 (88.9%)	1005 (12.3%)	Reference	–	Reference	–
	Schizoaffective disorder	520 (4.7%)	468 (90.0%)	34 (6.5%)	0.58 (0.41–0.81)	0.002	0.57 (0.40–0.80)	0.001
	Bipolar affective disorder	2495 (22.4%)	2078 (83.3%)	268 (10.7%)	0.85 (0.74–0.97)	0.015	0.82 (0.71–0.94)	0.005
Psychiatric admission	No	5963 (53.4%)	5022 (84.2%)	717 (12.0%)	Reference	–	Reference	–
	Yes	5199 (46.6%)	4768 (91.7%)	590 (11.3%)	0.91 (0.81–1.01)	0.078	0.93 (0.83–1.04)	0.198
Personality disorder	No	10,267 (92.0%)	8941 (87.1%)	1237 (12.0%)			Reference	–
	Yes	895 (8.0%)	849 (94.9%)	70 (7.8%)	0.91 (0.73–1.15)	0.444	0.77 (0.61–0.97)	0.028
Alcohol use disorder	No	10,158 (91.0%)	8825 (86.9%)	1133 (11.2%)	Reference	–	Reference	–
	Yes	1004 (9.0%)	965 (96.1%)	174 (17.3%)	1.6 (1.36–1.88)	<0.001	1.46 (1.23–1.72)	<0.001
Opioid use disorder	No	10,747 (96.3%)	9382 (87.3%)	1254 (11.7%)	Reference	–	Reference	–
	Yes	415 (3.7%)	408 (98.3%)	53 (12.8%)	1.82 (1.38–2.41)	<0.001	1.46 (1.08–1.97)	0.014
Other substance use disorder	No	9465 (84.8%)	8118 (85.8%)	1178 (12.4%)	Reference	–	Reference	–
	Yes	1697 (15.2%)	1672 (98.5%)	129 (7.6%)	1.13 (0.94–1.37)	0.192	0.97 (0.79–1.19)	0.789
Current smoker	No	1372 (12.3%)	0 (0.0%)	139 (10.1%)	Reference	–	Reference	–
	Yes	9790 (87.7%)	9790 (100%)	1168 (11.9%)	1.58 (1.33–1.89)	<0.001	1.49 (1.25–1.79)	<0.001

a Other ethnicity includes: Chinese, Any other ethnic group, Any other mixed background and White and Asian categories.

For females, life expectancy at birth was 67.6 years in current smokers (95% CI: 66.4–68.8) and 74.9 years in non-smokers (95% CI: 72.8–77.0), a difference of 7.3 years. In male participants, life expectancy at birth was 63.5 years in current smokers (95% CI: 62.5–64.5) and 68.5 years in non-smokers (95% CI: 64.4–72.6), a difference of 5.0 years. Life expectancy estimates according to diagnosis are provided in Supplementary Table 1. Due to small sample sizes, estimates for the schizoaffective disorder subgroups were not feasible and the estimates for other groups should be interpreted with caution.

The results of survival analyses using imputed data are described in Table 1, Table 2. In female participants, the age-adjusted HR for smoking was 1.51 (95% CI: 1.30–1.77, *p* < 0.001). In the second model, after adjustment for a range of demographic, socioeconomic and clinical variables it was 1.42 (95% CI: 1.21–1.66, *p* < 0.001). Older age-band and opioid use disorder were associated with increased mortality risk, while participants from Black African and Black Caribbean heritage had reduced risk. For males, the age-adjusted HR was 1.58 (95% CI: 1.33–1.89, *p* < 0.001). After adjustment for a broader range of confounders, the aHR was 1.49 (95% CI: 1.25–1.79, *p* < 0.001). In male participants, older age band, alcohol use disorder and opioid use disorder were associated with statistically significant increases in mortality risk, while Black African or Black Caribbean heritage, affective psychotic disorder diagnosis and personality disorder diagnosis were associated with reduced risk.

## 4 Discussion

Using a large clinical dataset, we found that people with a clinical diagnosis of schizophrenia, schizoaffective disorder or bipolar disorder who smoke cigarettes have a substantially lower life expectancy than non-smokers with the disorders. For women, the difference in life expectancy between current smokers and non-smokers was 7.3 years; for men, the gap was 5.0 years.

In 2012, the mid-point of this study, life expectancy in the UK general population was 82.8 years for women and 79.0 years for men (Office for National Statistics, 2017b). Similar estimates of life expectancy are found in each of four boroughs of South East London studied here (Office for National Statistics, 2015). If we compare the national life expectancy estimates (which contain both smokers and non-smokers) to the estimates obtained for participants in this study, the life expectancy gap was 15.1 years for female current smokers and 7.8 years for non-smokers. In men, the differences are 15.2 years for current smokers and 10.2 years for non-smokers. Smoking would therefore account for around 48% of the life expectancy gap in women and 33% in men. The difference in life expectancy was larger for women and would suggest that they may experience more harm from smoking. This is supported by observational research where smoking confers a higher risk of coronary heart disease in women (Huxley and Woodward, 2011). However, in the present study, after adjustment for other factors, the risks associated with smoking were similar across genders (aHRs: 1.42 vs. 1.49).

In the general population cigarette smoking is associated with a reduced life expectancy of around 7–10 years (Al Mamun et al., 2004; Doll et al., 1994; Sakata et al., 2012). In the study by Tam et al., the difference in life expectancy between current and never-smokers with self-reported serious psychological distress was 8.9 years in men and 10.2 years in women; the authors suggested that smoking may account for around two-thirds of the life expectancy gap (Tam et al., 2016). Other factors (such as medication, physical activity, substance use or suicide) may explain why, in this study, smoking accounted for a smaller proportion of the life expectancy gap.

Nevertheless, in our survival analyses, which account for a broad range of potential confounding factors, the risks associated with smoking were similar to co-morbid alcohol or opioid use disorders. Life expectancy in people with substance use disorders is also very low (Chesney et al., 2014), though a reasonable proportion of this difference may also be accounted for by smoking-related disease (Weinberger et al., 2016), which is consistent with our findings. The finding that cigarettes can confer mortality risks similar to heroin use or alcohol dependence could constitute an effective public health message.

The prevalence of smoking in this study was very high: 77.4% were classified as current smokers while only 15.9% were classified as non-smokers. The Adult Psychiatric Morbidity Survey (APMS) 2007 estimated that national smoking prevalence for people with ‘probable psychotic disorders’ was only 40% (Mcmanus et al., 2010). This difference is not due to geography, as smoking prevalence in South East London is similar to national rates (Department of Health and Office of National Statistics, 2017). Instead, it may be because the patients included in this study, who have had recent contact with mental health services or have been admitted to hospital, are more likely to be smokers. In a sample of ‘institutionalized’ patients with psychotic disorders from the 1996 APMS, the prevalence of smoking was 82% for non-affective psychoses and 78% for affective psychoses (Royal College of Physicians; Royal College of Psychiatrists, 2013).

Strengths of this study include the large size of the population included and the use of a clinical database which captures data from almost all NHS patients receiving secondary mental healthcare across South East London. The database was also linked to the NHS Care Records Service ensuring highly accurate mortality data. For diagnosis and smoking status, we used real-world clinical data enabling generalisation to routine clinical practice. However, the results may not be generalisable to patients who do not receive secondary mental healthcare or live outside of South East London, an ethnically diverse urban area with high levels of deprivation and socioeconomic inequality. The CRIS system extracts routinely recorded clinical data which are prone to entry errors by clinicians and entries are not intended for use in research. Data extraction errors also be present as the natural language processing application has a false positive rate of 7% (Wu et al., 2013). For each participant, smoking status may change over time and our study did not account for this.

Our results suggest that smoking may be one of the most important modifiable risk factors for mortality in patients with psychotic disorders seen in secondary mental health services. Since smoking is highly prevalent in these populations, it probably accounts for the largest proportion of premature mortality at a population level. Smoking cessation treatments, such as nicotine replacement therapy, varenicline and bupropion are well-established, safe and effective interventions (Gilbody et al., 2019; Peckham et al., 2017). In the past, concerns have been raised that such treatments may be unsafe for people with psychotic disorders, however, recent studies have challenged these (Anthenelli et al., 2016). Despite this evidence, the prescription of smoking cessation treatments and delivery of tailored smoking cessation programmes to people with psychosis are limited (Szatkowski and Aveyard, 2016). As a result, while rates of smoking in the general population are falling, the prevalence of smoking in people with severe mental illness remains stubbornly high (Szatkowski and McNeill, 2015).

## 5 Conclusions

In women with a diagnosis of schizophrenia, schizoaffective disorder, or bipolar affective disorder, current smokers have a life expectancy 7.3 years shorter than non-smokers. In men, current smokers have a life expectancy 5.0 years shorter. Since smoking is so prevalent in this population, these differences will account for a significant proportion of the cumulative years of life lost at a population level. If effective interventions to reduce smoking in patients with these disorders were implemented comprehensively, they would have a substantial impact on the size of the gap in life expectancy with the general population.

The following is the supplementary data related to this article.

Supplementary Table 1 Life expectancy at birth according to diagnostic group. Supplementary Table 1

## Transparency declaration

The lead author affirms that the manuscript is an honest, accurate, and transparent account of the study being reported; that no important aspects of the study have been omitted; and that any discrepancies from the study as planned have been explained.

## Additional statements

All researchers are independent from their funders. All authors had full access to all of the data (including statistical reports and tables) in the study and can take responsibility for the integrity of the data and the reliability of the data analysis.

## Contributions

EC designed of the study with input from DR, RP, CKC, PM and AM. HS completed the data extraction. EC completed the statistical analyses with support from CKC and SR. EC wrote the first draft of the manuscript. All authors contributed to and revised the final manuscript.

## Funding and sponsorship

This research was supported by the Biomedical Research Nucleus data management and informatics facility at South London and Maudsley NHS Foundation Trust, which is funded by the National Institute for Health Research (NIHR) Mental Health Biomedical Research Centre at 10.13039/100009362South London and Maudsley NHS Foundation Trust and 10.13039/501100000764King's College London and a joint infrastructure grant from 10.13039/501100000380Guy's and St Thomas' Charity and the 10.13039/100012176Maudsley Charity.

EC is funded by a 10.13039/501100000272National Institute for Health Research Doctoral Research Fellowship (NIHR300273).

RP has received support from a 10.13039/501100000265Medical Research Council (MRC) Health Data Research UK Fellowship (MR/S003118/1) and a Starter Grant for Clinical Lecturers (SGL015/1020) supported by the 10.13039/501100000691Academy of Medical Sciences, The Wellcome Trust, 10.13039/501100000265MRC, 10.13039/501100000274British Heart Foundation, 10.13039/501100000341Arthritis Research UK, the 10.13039/501100000395Royal College of Physicians and 10.13039/501100000361Diabetes UK.

C-KC was funded by the NIHR Specialist Biomedical Research Centre for Mental Health at the 10.13039/100009362South London and Maudsley NHS Foundation Trust and Institute of Psychiatry, Psychology, and Neuroscience, 10.13039/501100000764King's College London. C-KC had received research funding from recent 10.13039/100004337Roche cooperation (negative symptoms and the other dementia studies). No support from any other organisation for the submitted work and no other relationships or activities that could appear to have influenced the submitted work as declared.

DR and AM are part funded by the 10.13039/100006662NIHR Collaboration South London (NIHR ARC South London) at King's College Hospital NHS Foundation Trust. AM is an NIHR Senior Investigator. The views expressed are those of the authors] and not necessarily those of the NIHR or the Department of Health and Social Care.

## Role of funders and sponsors

The views expressed are those of the authors and not necessarily those of funding organisations. The funders had no role in the design and conduct of the study; extraction, management, analysis, and interpretation of the data; preparation, review, or approval of the manuscript; and decision to submit the manuscript for publication.

## Ethical approval

The CRIS data resource received ethical approval as an anonymized dataset for secondary data analyses from Oxfordshire REC C (Ref: 08/H0606/71+5).

## Data sharing

Patient-level data is not freely available without local authorization. Syntax for data preparation and modelling in Stata is available from the corresponding author on request.

## Declaration of competing interest

None.
