# Supplementary material for: The impact of cigarette smoking on life expectancy in schizophrenia, schizoaffective disorder and bipolar affective disorder: An electronic case register cohort study
Source: Schizophr Res. 2021 Dec;238:29–35. doi: 10.1016/j.schres.2021.09.006 (PMC8653908; doi:10.1016/j.schres.2021.09.006)
Supplement: Supplementary Table 1 — Life expectancy at birth according to diagnostic group. [file mmc1.docx]

| Supplementary Table 1. Life expectancy at birth according to diagnostic group | | | | | | | | | | |
| --- | --- | --- | --- | --- | --- | --- | --- | --- | --- | --- |
|  |  | Smokers |  |  |  | Non-smokers | |  |  |  |
| Gender | Diagnosis | Sample Size (n) | Population-years | Deaths (n) | Life expectancy (years) | Sample Size (n) | Population-years | Deaths (n) | Life expectancy (years) | Difference in life expectancy (years) |
| Male | SMI* | 9790 | 74842 | 1235 | 63.5 (62.5 - 64.5) | 1372 | 10063 | 148 | 68.5 (64.4 - 72.6) | 5.0 |
|  | Schizophrenia | 7244 | 56659 | 958 | 62.9 (61.8 - 64.0) | 903 | 6843 | 102 | 65.9 (59.5 - 72.3) | 3.0 |
|  | Bipolar affective disorder | 2078 | 14423 | 243 | 64.4 (62.4 - 66.5) | 417 | 2789 | 42 | 72.1 (67.5 - 76.7) | 7.7 |
|  |  |  |  |  |  |  |  |  |  |  |
| Female | SMI* | 6927 | 49458 | 830 | 67.6 (66.4 - 68.8) | 2066 | 14512 | 221 | 74.9 (72.8 - 77.0) | 7.3 |
|  | Schizophrenia | 3753 | 27781 | 539 | 66.9 (65.3 - 68.5) | 1142 | 8388 | 141 | 75.8 (73.4 - 78.3) | 8.9 |
|  | Bipolar affective disorder | 2766 | 18427 | 243 | 68.2 (66.2 - 70.1) | 811 | 5245 | 65 | 73.7 (70.2 - 77.2) | 5.5 |
| *SMI sample sizes include 520 males and 521 females  with schizoaffective disorder | | | | | | | | | | |
